# Hybrid Genome Assembly of a Neotropical Mutualistic Ant

**DOI:** 10.1093/gbe/evz159

**Published:** 2019-07-22

**Authors:** Juliane Hartke, Tilman Schell, Evelien Jongepier, Hanno Schmidt, Philipp P Sprenger, Juraj Paule, Erich Bornberg-Bauer, Thomas Schmitt, Florian Menzel, Markus Pfenninger, Barbara Feldmeyer

**Affiliations:** 1 Senckenberg Biodiversity and Climate Research Centre, Frankfurt am Main, Germany; 2 Institute of Organismic and Molecular Evolution (iOME), Johannes Gutenberg University, Mainz, Germany; 3 LOEWE Centre for Translational Biodiversity Genomics (LOEWE-TBG), Frankfurt am Main, Germany; 4 Molecular Evolution and Bioinformatics Group, Institute for Evolution and Biodiversity, Westfälische Wilhelms-Universität, Münster, Germany; 5 Vector Genetics Laboratory, Department of Pathology, Microbiology and Immunology, School of Veterinary Medicine, University of California, Davis; 6 Department of Animal Ecology and Tropical Biology, University of Würzburg, Biocentre – Am Hubland, Germany; 7 Department of Botany and Molecular Evolution, Senckenberg Research Institute and Natural History Museum, Frankfurt am Main, Germany

**Keywords:** cuticular hydrocarbons, assembly, MinION, formicine, elongase, desaturase

## Abstract

The success of social insects is largely intertwined with their highly advanced chemical communication system that facilitates recognition and discrimination of species and nest-mates, recruitment, and division of labor. Hydrocarbons, which cover the cuticle of insects, not only serve as waterproofing agents but also constitute a major component of this communication system. Two cryptic *Crematogaster* species, which share their nest with *Camponotus* ants, show striking diversity in their cuticular hydrocarbon (CHC) profile. This mutualistic system therefore offers a great opportunity to study the genetic basis of CHC divergence between sister species. As a basis for further genome-wide studies high-quality genomes are needed. Here, we present the annotated draft genome for *Crematogaster levior* A. By combining the three most commonly used sequencing techniques—Illumina, PacBio, and Oxford Nanopore—we constructed a high-quality de novo ant genome. We show that even low coverage of long reads can add significantly to overall genome contiguity. Annotation of desaturase and elongase genes, which play a role in CHC biosynthesis revealed one of the largest repertoires in ants and a higher number of desaturases in general than in other Hymenoptera. This may provide a mechanistic explanation for the high diversity observed in *C. levior* CHC profiles.

## Introduction

The genomic basis of chemical communication is still mostly unknown, despite its importance in animal behaviour. A prime example are social insects, in which cuticular hydrocarbons (CHCs) represent the most important means of communication and facilitate the functioning of complex social organization. They enable the expression and recognition of various attributes, such as species and nest-mate status, caste, sex, and fertility (Lahav et al. 1999; Dietemann et al. 2003; Leonhardt et al. 2016). CHCs cover the cuticle of all insects and originally evolved as a protection against desiccation (Blomquist and Bagnères 2010; Menzel et al. 2018). Because of their function in both ecological adaptation and mate signaling, they were proposed as drivers of speciation (Thomas and Simmons 2009; Smadja and Butlin 2009; Chung and Carroll 2015), and thus may have driven the high diversity witnessed today in social insects.

One of the most successful families of social insects is ants with ∼13,000 recognized species (Chomicki and Renner 2017). They occur in virtually all terrestrial habitats, barring the polar regions, and evolved a striking diversity in life-history traits, morphology and behavior. This diversity, however, is not reflected in the number of published genomes so far (*n* = 19).

The Neotropical ant species *Crematogaster levior* and *Camponotus femoratus* are representative of the remarkable diversity within this family, as they mutualistically share a nest, a so-called ant garden (Davidson 1988). Obligate mutualisms that are characterized by a benefit for both partners are rare. Here, *Crematogaster* benefits from strong defense capabilities of *Camponotus*, whereas the latter benefits from *Crematogasters* efficiency in finding resources (Vantaux et al. 2007). Both species show unusually high diversity in their CHC profiles (Menzel et al. 2014) that were now shown to represent cryptic species (Hartke et al. 2019). This mutualism therefore offers the unique chance to study the underlying genomic basis of CHC complexity and their putative function in species divergence in two closely related species. Here, we present the first annotated draft genome for one of the cryptic *Crematogaster* species, *C.levior* A, and compare the number of genes with putative function in communication to other available ant and hymenopteran genomes.

## Materials and Methods

### Sample Collection and Sequencing

Specimens for sequencing were collected from a single nest in, French Guiana (4°33′14.5″N 52°09′02.4″W), in September 2016. The ants were stored in 96% ethanol until DNA isolation. We followed a hybrid approach, acquiring sequences from three different sequencing platforms. To obtain sufficient amounts of DNA for sequencing, we pooled 70 larvae for HiSeq 2000 (Illumina Inc, CA, USA) paired-end sequencing, 110 larvae for two SMRT cells on PacBio Sequel (Pacific Biosciences, CA, USA) and >300 larvae for a total of six sequencing runs on an Oxford Nanopore Technologies (ONT), UK, MinION. Illumina and PacBio sequencing were conducted at the Beijing Genomics Institute (BGI), Hong Kong, and Oxford Nanopore sequencing inhouse.

DNA for Illumina sequencing was isolated with the DNeasy Blood and Tissue Kit (Qiagen), following manufacturer's instructions. DNA isolation and library preparation for PacBio sequencing were partly conducted by BGI, Hong Kong, plus additional DNA isolated from our lab by DNeasy Blood and Tissue kit. We constructed four different libraries for a total of six ONT MinION runs, for which we tested different DNA isolation and library preparation protocols. We isolated two DNA samples following the Qiagen Blood and Tissue Protocol, and two samples following Urban et al. (2015 preprint), which is optimized for long high molecular weight DNA. The library preparation was conducted three times following the latest ONT protocol and once using the Urban et al. (2015 PREPRINT) protocol (details in supplementary information M1 and supplementary table S1, Supplementary Material online).

For transcriptome sequencing, specimens of the same nest were freeze killed at –80 °C. We isolated RNA from different worker stages (newly emerged and old workers, young and old worker pupae). We furthermore isolated RNA from eggs of an additional colony. Extraction protocol followed Alleman et al. (2018). Sequencing on a HiSeq 2000 was conducted by BGI, Hong Kong. For extraction, pre-assembly processing and assembly protocol please refer to supplementary M2, Supplementary Material online. We furthermore assembled transcriptomes of the sister species, *C. levior* B (BioProject PRJNA540400).

### Assembly Strategy

Illumina reads were quality-trimmed and filtered for adapter sequences with the BBDuk algorithm from BBMap v36.92 (Bushnell 2014), screened for contamination using FastQ Screen v0.10.0 (Wingett et al. 2018), and filtered for mtDNA with BBDuk. Before and after every processing step, read quality was checked with FastQC v0.11.3. PacBio reads were quality corrected with Proovread v2.14.0 (Hackl et al. 2014), using the Illumina read set to obtain high-quality reads. MinION reads were base called and quality-filtered with the Nanopore basecaller Albacore v2.0 (ONT, UK) and subsequently filtered for mtDNA with BBDuk. For more details see supplementary material M3, Supplementary Material online.

The Illumina read set was assembled with SPAdes v3.10.0 (Bankevich et al. 2012) using default settings, and the resulting assembly was triplicated to a coverage of 3× to be included by the algorithm of the next assembler. This set of contigs, together with ONT and PacBio reads was assembled with the long-read assembler Ra (github.com/rvaser/ra; commit ID: 65bedfe). The resulting assembly was scaffolded with SSPACE-LongRead v1.1 (Boetzer and Pirovano 2014) using ONT and PacBio long reads (see supplementary methods M4, Supplementary Material online). We assessed repeat content within our Illumina read set using RepeatExplorer (Novák et al. 2013), and checked for the completeness of gene space with BUSCO v2.0 (Simão et al. 2015) with the provided database for hymenopteran orthologous genes.

### Genome Size Estimation

We estimated genome size by dividing the total number of nucleotides used in the Illumina assembly by the peak coverage resulting from mapping those reads back to the assembly (Schell et al. 2017). Additionally, genome size was also estimated using flow cytometry with three individuals of *C. levior* A, and *Glycine max* cv. Polanka as an internal standard (see supplementary methods M5, Supplementary Material online).

### Annotation Strategy

Before annotation, we masked all regions that were covered only by uncorrected PacBio or MinION reads with bedtools *maskfasta* (Quinlan and Hall 2010), to base gene predictions only on high-quality information throughout the assembly. Gene annotation was conducted using the MAKER2 pipeline v2.31.8 (Holt and Yandell 2011). As evidence, we used transcriptomes from *C. levior* A; additional ESTs from the sister species, *C. levior* B (worker; BioProject PRJNA540400; see Sprenger et al. in prep); ab initio models from SNAP v2006-07-28 (Korf 2004), Augustus v3.2.2 (Stanke et al. 2006), and GeneMark v4.32 (Lomsadze et al. 2005); and the repeat library. As protein homology evidence, we used the SwissProt Database (accessed September 22, 2017) and an annotated protein set of *Cardiocondyla obscurior*, which is the most closely related ant species with a published genome (Schrader et al. 2014). For a more detailed protocol refer to supplementary M6, Supplementary Material online. Moreover, we manually annotated elongases and desaturases (supplementary methods M7, Supplementary Material online). We also searched for elongases and desaturases in 43 annotated Hymenoptera genomes via a blastp v2.5.1 (Camacho et al. 2009) and PfamScan v1.6 (Punta et al. 2012) workflow (see supplementary methods M8, Supplementary Material online).

## Results and Discussion

### Genome Sequencing and Assembly

An overview of raw sequences obtained from each sequencing strategy and number of trimmed reads can be found in supplementary table S3, Supplementary Material online. Genome size, assessed by the peak coverage approach (Schell et al. 2017), was estimated to be 355.52 Mbp. This estimate is at the higher end but still within range compared with other ant genomes (supplementary table S10, Supplementary Material online). Genome size (2C-value) was also estimated by flow cytometry (see supplementary M4, Supplementary Material online). When correcting the original *G. max* calibration (Doležel et al. 1994) for the newest human reference genome assembly (GRCh38.p13), the 2C value corresponds to 409.96 Mbp (1 pg = 978 Mbp, Doležel et al. 2003), which is within range of previously reported estimates, although significantly larger than estimates for the same genus (*Crematogaster hespera*: 275.9 Mbp; Tsutsui et al. 2008). The difference in size estimates from flow cytometry and peak coverage might be explained by the loss of sequences during library preparation. Regions in the DNA with long stretches of repeats are prone to harbor breakage points or form secondary structures, such as hairpins (De Bustos et al. 2016), that hinder sequencing in those regions and thereby lead to faulty coverage estimations by read distribution.

Assembly and scaffolding resulted in 1,523 scaffolds with a N50 length of 383,244 bp and a total length of 326.2 Mbp (peak coverage: 92% of the estimated size, flow cytometry: 80% of the estimated size). To assess gene-space completeness of the draft genome, BUSCO v2.0 was used with the provided Hymenoptera data set of core orthologues, of which 98.0% could be retrieved (*N* = 4,415; complete: 95.9%, fragmented: 2.1%, missing: 2.0%), suggesting a high level of completeness and contiguity of coding regions.

Approximately 12.2% of the genome assembly consist of repeats, with the largest portion being labeled as unclassified (65%), followed by LINEs and LTRs (both 11%) (supplementary fig. S1, Supplementary Material online). Most ant genomes sequenced so far, have higher reported repeat contents (mean = 24%; supplementary table S10, Supplementary Material online). Especially when regarding the fact that up to 20% of the estimated genome size could not be assembled, which is most likely due to repeat regions, the estimates by RepeatExplorer (10.5%) and RepeatModeler (3.2%) seem too low, which is in line with the above given reasoning of either break points and/or secondary structures of the DNA in repeat regions, which leads to lower representation of these regions in the sequences used for assembly. Backmapping rates are very high with over 96% for each sequencing method (supplementary table S11, Supplementary Material online), indicating that over 95% of the actually sequenced reads are represented in the final assembly.

### Comparison of Assembly Strategies

We used different combinations of our read data as input for Ra and are thus able to compare the influence of single read types on the accuracy and contiguity of the assembly (table 1). From all single read type assemblies, the one from uncorrected PacBio reads seemed to be the most continuous, but it lacks in accuracy with 0% of BUSCO orthologues found. Prior correction with Illumina data improved the assembly immensely (90.5% found orthologues). When combining corrected PacBio reads with the Illumina assembly, quality metrics improved further, albeit only slightly. The MinION only assembly also lacked in accuracy and compared with the PacBio assembly, also in completeness (32% of final assembly length). A combination of the corrected PacBio reads with MinION reads lead to a substantial drop in accuracy (11.7% found orthologues) compared with the assembly without MinION reads. By combining all three read types, we obtained the best results in terms of length and accuracy (98% of orthologues). Especially, when comparing this 3-way assembly to the one lacking MinION reads, the difference in contiguity and accuracy is striking. N50 increased by >120 kbp and we found 6% more BUSCO orthologues. This shows that even a coverage of MinION reads as low as 9x can significantly increase assembly contiguity, although this only held true when Illumina reads were added.

**Table 1:** Overview of Different Assembly Approaches for *Crematogaster levior* A Using Different Combinations of Illumina, MinION, and PacBio Reads

Read Type	#Contigs	N50	Length [Mbp]	Recovered BUSCO [%]
Illumina	52,838	15,083	259.9	95.4
MinION	3,420	39,345	114.3	2.8
PacBio	3,270	142,016	319.9	0
PacBio polished	3,615	104,646	298.8	90.5
MinION & PacBio	1,898	361,377	326.6	10.1
MinION & PacBio polished	2,207	260,013	325.9	11.7
PacBio polished & Illumina (3×)	3,311	120,772	299.9	92.4
PacBio polished & MinION & Illumina (3×)	2,298	242,096	324.2	98.0

Note.—Illumina (3×): Illumina reads were added as triplicates to the hybrid assembly. All assemblies were conducted with Ra, except for the Illumina only assembly that was assembled using Spades.

Finally, we analyzed which fraction of the final assembly was uniquely covered by single read types (supplementary table S12, Supplementary Material online). Only 1.05% of the draft was covered solely by Illumina reads. For PacBio, the percentage was higher with 2.33%, including 1.31% of the assembly that was covered by uncorrected PacBio reads only. Genome positions that were only covered by MinION reads made up 2.42% of the final assembly.

### Annotation Report

MAKER2 annotation resulted in 17,855 genes that comprise 31% of the assembly space (table 2). The number of annotated genes is within the same range as other annotated ant genomes (supplementary table S10, Supplementary Material online). Using a blastp search against the NCBI nonredundant invertebrate database (accessed March 2019), we were able to retrieve 14,713 genes, indicating 3,142 putative taxonomically restricted genes within *C. levior* A. This number is lower than previously found in other Hymenoptera species (Simola et al. 2013), however, the number of available genomes and thereby the number of similar genes increased in the meantime, which may explain the discrepancy. Mean GC content genome-wide (36%), within exons (43%) and within introns (30%) was similar to other reports on invertebrates (Jiang et al. 2014).

**Table 2:** Genome Statistics of Final Assembly, Containing All Three Read Types, After Scaffolding

Genome Statistics After Scaffolding	
Parameter	Value
#Scaffolds	1,523
Assembly length	326.2 Mbp
N50	383,244 bp
Gaps (N)	0.63%
BUSCO orthologous genes present	98.00%
#Genes	17,855
Gene space (UTR, exons, introns, etc.)	103 Mbp (31.66% of assembly)
Mean distance between genes	6,479 bp
#Exons	117,323
Exon space	36 Mbp (11.27% of assembly)
Exons/gene	6.6

### Comparison of Gene Families

**Fig. 1.**
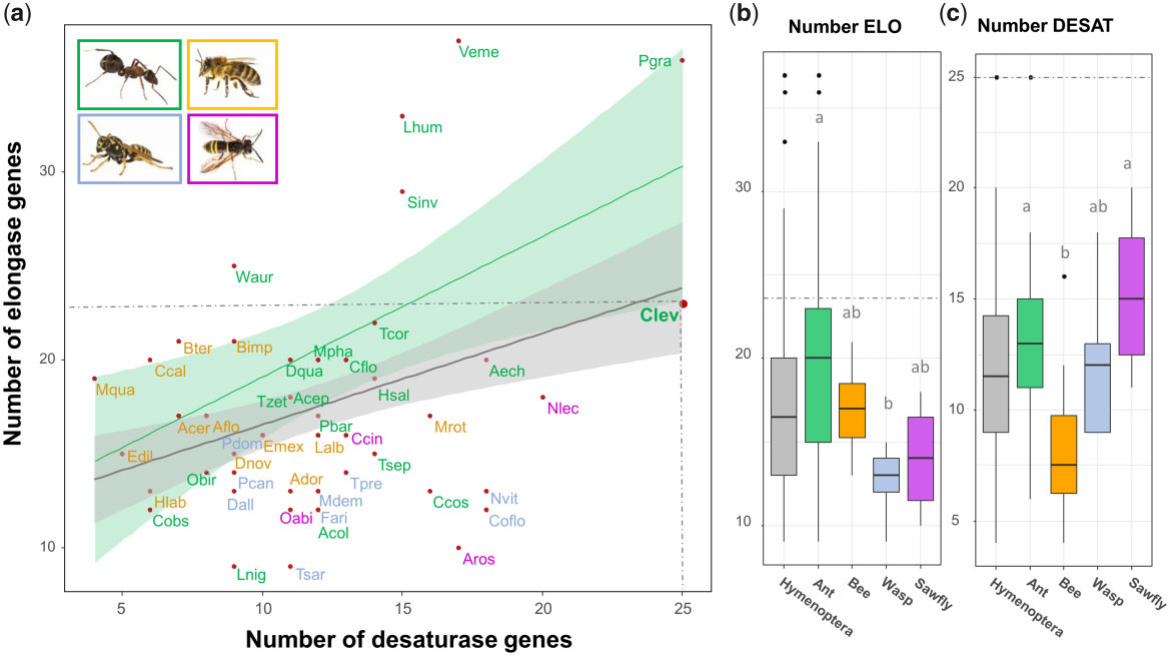
—(*a*) Relationship between the number of elongase and desaturase genes across 48 hymenopterans (see also supplementary table S8, Supplementary Material online). The different colors depict the different families (green: ants, yellow: bees, red: wasps, purple: sawflies). Similarly, the green regression was calculated based on ants, whereas the gray regression was calculated based on all Hymenoptera. Pictures show exemplary species for each family (ant, bee, wasp [all Barbara Feldmeyer], sawfly [Alex Hyde]). Comparison of the number of (*b*) elongases and (*c*) desaturases across hymenopteran families. Different letters indicate significant difference in number of genes (significance level: *P* < 0.05; One-way ANOVA, Tukey’s HSD, supplementary table S9, Supplementary Material online). The dotted lines indicate the number of genes found in *Crematogaster levior* A.

Elongases and desaturases are among the gene families that play key roles in the biosynthesis of CHCs (Falcón et al. 2014). To detect differences within gene family sizes between closely related species, high quality genomes are needed. By manually annotating elongases and desaturases, we moreover tested the contiguity of our assembly, and found 23 elongases and 25 desaturases in the *C. levior* A draft genome (supplementary table S6, Supplementary Material online). We compared these values to 47 other hymenopteran draft genomes (fig. 1, supplementary table S8, Supplementary Material online) and found significant differences between groups (elongases: *P* = 0.015; desaturases: *P* = 0.008, one-way ANOVA). Ants had significantly more elongases than wasps (fig. 1*b*, supplementary table S9, Supplementary Material online) and bees had significantly fewer desaturases than ants and sawflies (fig. 1*c*, supplementary table S9, Supplementary Material online). Among all analyzed species, *C. levior* A and *Pseudomyrmex gracilis* had the highest number of desaturases (mean number in ants: 13.7, Hymenoptera: 12.0). In line with increased chain elongation in *C. levior* A (Sprenger PP, Hartke J, Feldmeyer B, Orivel J, Schmitt T, Menzel F, submitted), their number of elongases was higher than the general mean in ants (20.6, Hymenoptera: 17.5). On the one hand this increased number of elongases and desaturases may be a major part of the genomic basis of high intraspecific CHC variation reported in *C. levior* (Menzel et al. 2017), on the other hand it might be reflective of a highly contiguous and complete assembly within coding regions.

## Conclusion

Here, we present the annotated draft genome of *C.levior* A. By using a hybrid assembly approach encompassing three different sequencing techniques, and by combining high-quality short reads with long reads, we were able to produce a high-quality de novo ant genome assembly. Even rather low coverages of long reads significantly increased accuracy and contiguity and are a good and cost-effective way to obtain high-quality draft genomes. A comparison to other Hymenoptera yielded strong differences between species in the total number of desaturase and elongase genes. Among all analyzed species, *C. levior* A (together with *P. gracilis*) showed the highest number of desaturases, which may be reflective of their high intraspecific diversity in CHC profiles.

## Supplementary Material

Supplementary data are available at *Genome Biology and Evolution* online.

## Supplementary Material

evz159_Supplementary_Data

## Notes

**Data deposition:** This project has been deposited at ENA under the accession PRJEB32544.
