# Supplementary material for: Hybrid Genome Assembly of a Neotropical Mutualistic Ant
Source: Genome Biol Evol. 2019 Jul 22;11(8):2306–11. doi: 10.1093/gbe/evz159 (PMC6735702; doi:10.1093/gbe/evz159)
Supplement: evz159_Supplementary_Data [file evz159_supplementary_data.zip › Supplement.docx]

Supplement

Supplementary methods

M1: ONT MinION sequencing protocol

Sequencing was conducted using six flowcells and four different DNA isolations and library preparations. The first isolation and library preparation followed standard DNeasy Blood and Tissue kit (Qiagen) and standard Nanopore protocol for 2D reads. The second isolation and library preparation were done following the protocol of Urban et al. (2015 Preprint) specifically designed to obtain long reads. For the third run, we used DNA isolated with the Urban et al. protocol and prepared the library following standard MinION 1D protocol. The fourth run was prepared as the first one, but with the MinION 1D protocol, and we used library left over from the first run to top up the flowcell during sequencing. For the fifth run, we used libraries prepared for the third and fourth run, and for the sixth run, we used a left-over library from the third run.

**Table S1: Overview of Oxford Nanopore MinION sequencing runs and their output**

|  | **DNA isolation** | **Library prep** | **Base pairs** | **Reads** | **N50 [bp]** | **Max length [bp]** |
| --- | --- | --- | --- | --- | --- | --- |
| **Run 1** | Qiagen | Nanopore 2D | 479,650,038 | 373,578 | 2,674 | 32,955 |
| **Run 2** | Urban et al. | Urban et al. | 13,110,799 | 5,219 | 4,944 | 69,407 |
| **Run 3** | Urban et al. | Nanopore 1D | 274,508,112 | 47,050 | 10,304 | 133,542 |
| **Run 4** | Qiagen | Nanopore 1D + Run 1 | 1,080,604,289 | 653,752 | 2,156 | 73,473 |
| **Run 5** |  | Run 3 + 4 | 800,540,339 | 316,154 | 4,036 | 277,903 |
| **Run 6** |  | Run 3 | 696,465,807 | 120,491 | 9,497 | 445,755 |

M2: Transcriptomes – extraction, processing, and assembly

Frozen samples of different worker stages (eggs, young pupae, old pupae, newly emerged worker and older worker) were ground in Trizol until homogenized. After adding 200 µl of chloroform the sample was shaken vigorously for 15 seconds. The mixture was centrifuged for 15 min at 4 °C at 11,600 rcf. The resulting aqueous phase was transferred to a new RNase-free tube and precipitated by adding 0.5 volume of absolute ethanol. The solution was pipetted four times to mix and then transferred to a RNeasy mini-spin column (Qiagen). The following steps follow step three onwards of the RNeasy Clean-Up manual (Qiagen). The column was centrifuged for 30 seconds at 10,000 rcf. RW-Buffer was added, and the column was centrifuged again. A wash step with RPW buffer followed and the column was then placed in a new 1.5 ml collection tube. For elution, 30 µl RNase free water was pipetted directly onto the column and incubated for 1 min at room temperature. A final centrifugation step for 1 minute at 10,000 rcf followed.

Transcriptomes were sequenced by BGI, Hong Kong, on an Illumina HiSeq 4000 to obtain paired-end reads with an insert size of 200 bp. The resulting sequences plus additional sequences from the same species (Sprenger et al. in prep) were trimmed using the autotrim v0.5 wrapper script based on Trimmomatic (Bolger et al. 2014; Waldvogel et al. 2018) (Trimmomatic-0.36/adapters/TruSeq3-PE-2.fa:2:30:10 TRAILING:3 SLIDINGWINDOW:4:15 -nok -rn). The resulting reads were quality checked with FastQC v0.11.3 (Andrews 2010) and assembled with Trinity v2.0.6 (Grabherr et al. 2013) (--seqType fq --min_contig_length 300 ‑‑full_cleanup --no_bowtie). We furthermore obtained sequences of the cryptic sister species, *Crematogaster levior* B (Sprenger et al. in prep), and followed the same assembly protocol with Trinity.

M3: Preparing of sequences prior to assembly

*M3.1 Quality processing Illumina:*

First, Illumina adapters were trimmed using BBDuk (part of BBMap 36.92) (minlen=25 qtrim=rl trimq=20 ktrim=r k=25 mink=11 ref=/opt/bbmap/resources/adapters.fa hdist=1 tpe tbo). Then, BBDuk was used to discard bases below a quality threshold of 20 (minlen=25 qtrim=rl trimq=20 k=25 mink=11). Only reads that were still properly paired after these filtering steps were retained, meaning if one read of the pair was removed during trimming, the other one was removed as well. We checked our read set for contaminations with FastQ Screen 0.10.0 (Wingett et al. 2018) using a custom database (Table S2). The mitochondrial genome was assembled with MITObim (Hahn et al. 2013) with default parameters by using the mitochondrial genomes of *Atta cephalotes (HQ415764.1)*, *Pristomyrmex punctatus (NC_015075)* and *Vollenhovia emeryi* (NC_030176.1) as a reference. The resulting assembly was then used in BBDuk (k=41 hdist=2) to filter our Illumina read set for mitochondrial DNA before the assembly.

**Table S2:** Reference species and databases used for contamination filtering with FastQ Screen

| **Species / database** | **Accession numbers / version** |
| --- | --- |
| *Homo sapiens* | GCF_000001405.35 |
| *Mus musculus* | GCF_000001635.25 |
| *Escherichia coli* | U00096.2 |
| PhiX | NC_001422.1 |
| *Ananas comosus* | GCA_001540865.1 |
| *Boea hygrometrica* | GCA_001598015.1 |
| *Orchesella cincta* | GCA_001718145.1 |
| *Spirodela polyrhiza* | GCA_000504445.1 |
| *Wolbachia* | GCF_000204545.1 |
| Deconseq bact/vir | V0.4.3 |

*M3.2 Quality processing MinION Nanopore reads:*

MinION reads were basecalled using the Nanopore basecaller Albacore with standard settings. This resulted in a total of 3.34 gbp distributed on 1.51 million reads. N50 was highly variable between runs and ranged from 2.1 kb to 10.3 kb. As for Illumina reads, MinION reads were filtered for mitochondrial DNA using BBDuk and subsequently for adapters used during sequencing (k=17 mink=11 hdist=2 ref=hairpin.fasta).

*M3.3 Quality processing PacBio reads:*

Sequencing with PacBio Sequel results in reads that were already filtered for adapters. Since these reads do not have any quality scores, we did not perform any filtering steps regarding quality as we did for Illumina reads. Instead, we used Proovread (Hackl et al. 2014) with standard settings to correct the data set with Illumina reads, that are less error-prone. For the final hybrid assembly, we used quality corrected, but untrimmed PacBio reads. This means that reads that could only partly be corrected by Illumina reads retained their original length and were only corrected for those positions with enough Illumina coverage.

**Table S3**: Sequence data before and after processing steps

|  | **Before processing** | | **after processing** | |
| --- | --- | --- | --- | --- |
|  | Reads | Nucleotides | Reads | Nucleotides |
| **Illumina** | 256,243,982 | 35,057,868,150 | 233,435,232 | 30,980,846,066 |
| **PacBio** | 1,138,469 | 6,653,714,229 | 656,310 | 4,211,462,945 |
| **MinION** | 1,516,244 | 3,344,879,393 | 1,483,045 | 3,204,107,205 |

M4: Assembly

1. Illumina only

For the Illumina only assembly we used SPAdes v3.10.0 (Bankevich et al. 2012)(--only_assembler 12), and afterwards retained only those contigs that were more than 500 bp long.

b) Hybrid-assembly

For the combined assembly we used the Illumina assembly that we duplicated to an artificial coverage of 3x to represent long reads, the corrected PacBio reads and the filtered MinION reads. We used Ra (https://github.com/rvaser/ra) with default options as assembler.

c) Scaffolding

The resulting assembly was scaffolded with the SSPACE-LongRead.pl v1.1 (Boetzer & Pirovano 2014) script using MinION and quality-corrected but untrimmed PacBio reads and default options.

M5: Estimating genome size

a) Peak coverage

We used the total number of trimmed nucleotides that were used for the Illumina assembly (38,396,416,216) and divided this by the maximum peak coverage of the per-position coverage frequency distribution (108) as an approximation of genome size. This method assumes an evenly distributed sequencing coverage throughout the genome (Schell et al. 2017).

b) Flow cytometry

Genome size (2C-value; Greilhuber et al. 2005) was estimated by flow cytometry using 3 individuals of *Crematogaster levior* and the Partec CyFlow Space (Partec, Münster, Germany) equipped with a green solid-state laser (Partec, 532 nm, 30 mW). Sample preparation followed two-step Otto protocol (Otto 1990), with an internal standard *Glycine max* cv. Polanka (2C = 2.50 pg; Doležel et al. 1994). Either the whole *C. levior* body or dissected head was mixed with ca. 1 cm^2^ leaf of an internal reference standard and homogenized with a razor blade in a Petri dish containing 1 ml of ice-cold Otto I buffer (0.1 M citric acid, 0.5% Tween 20; Otto 1990). The suspension was filtered through a 42-μm nylon mesh and incubated for approximately 15 min at room temperature. The staining solution consisted of 1 ml of Otto II buffer (0.4 M Na_2_HPO_4_·12 H_2_O), β-mercaptoethanol (final concentration of 2 μl/ml), intercalating fluorochrome propidium iodide (PI) and RNase IIA (both at final concentrations of 50 μg/ml). Fluorescence intensities of 15,000 particles (nuclei) were recorded for three replicates. Sample/standard ratios were calculated from the means of the sample and standard fluorescence histograms, and only histograms with coefficients of variation <3% for the G0/G1 sample peak were considered. The genome size was calculated by multiplying the sample/standard ratios with the genome size of the internal standard. Additionally, the genome size was corrected by approximately 4.41 %. This is the difference in assigned genome size of human male leukocytes (2C = 7.00 pg = 6,846 Mbp; Doležel et al. 1994, 2003) in the original calibration of *G. max* cv. Polanka and the most recent human reference genome assembly (GRCh38.p13; 2C = 6,544.234 Mbp; GenBank assembly accession: GCA_000001405.28).

M6: Annotation

RepeatModeler v1.0.4 (Smit et al. 2008) was run with default parameters on the final, scaffolded genome assembly, resulting in a repeat library that was used by MAKER2 (Holt & Yandell 2011) to mask repeat regions in the genome. For annotation, we used the MAKER2 v2.31.8 pipeline, which is not a gene prediction tool as such but a pipeline, that comprises multiple *ab initio* gene prediction tools such as SNAP (Korf 2004), Augustus (Stanke et al. 2006) and GeneMark (Lomsadze et al. 2005), and uses evidence alignments to find the best suited gene model for a given location.

We first created an Augustus (v3.2.2) species model based on the assembly, ESTs from the same species, and annotations of conserved orthologous genes computed with BUSCO v2.0 (insect dataset, option --long; Simão et al. 2015). We also created a SNAP v2006-07-28 (Korf 2004) model based on a CEGMA v2.5 (Parra et al. 2007) run on the assembly, by using the cegma2zff script (MAKER2) and the SNAP scripts fathom (fathom genome.ann genome.dna -categorize 1000 && fathom -export 1000 -plus uni.ann uni.dna), forge (export.ann export.dna) and hmm-assembler.pl. GeneMark v4.32 (Lomsadze et al. 2005) was used in self-training mode (--ES) on the assembly.

As input for MAKER we used the assembly; the Augustus model; the HMM models from SNAP and Genemark; ESTs from the same species, *C. levior* A; ESTs from *C. levior* B as alternative evidence; the Swiss-Prot/UniProt Database (accessed September 22^nd^, 2017); annotated proteins from *Cardiocondyla obscurior*; and the repeat library. Protein2genome and est2genome options were switched off and only proteins >30 AA were retained.

After the first iteration, the script gff3_merge was used to obtain a gff3 file and from this we built another Hidden Markov model with SNAP, as specified above. The SNAP model was converted to gff3 format using zff2gff2.pl (genome.ann | perl -plne 's/\t(\S+)$/\t\.\t$1/’) and the Augustus model was retrained based on this file. Then MAKER2 was run for a second iteration as specified above, but with updated SNAP and Augustus models. For a second retraining, we repeated those steps and ran MAKER2 for a third time.

To assess completeness of our annotated proteome, we ran DOGMA (Dohmen et al. 2016), which uses the Pfam v31 (El-Gebali et al. 2019) database of conserved protein domains. In addition, DOGMA was also run on other annotated ant genomes available at the time of the analysis (Table S10).

M7: Manual annotation of specific gene families in *C. levior*

Elongase (ELO), desaturase (DESAT), odorant binding protein (OBP), gustatory receptor (GR), chemosensory protein (CSP) and odorant receptor (OR) gene families were manually annotated using a two-pass tblastn/Exonerate (Slater & Birney 2005) + GeMoMa (Keilwagen et al. 2016) + Web Apollo (Lee et al. 2013) workflow. In the first round, manually annotated (i.e. high quality) target genes of *Temnothorax longispinosus* (Kaur et al. 2019) were blasted against the *C. levior* A assembly using tblastn v2.2.30 (e-value=1e-03). Target genes were annotated with Exonerate v2.2.0 (parameter settings: --model protein2genome --percent 50) in those genomic regions with blast hits. In parallel, GeMoMa v1.4.2 was used to annotate target genes, based on the annotation of eight reference ant genomes (Table S4) as well as the worker RNAseq libraries of *C. levior* A for intron predictions. All protein sequences of the reference species were annotated with PfamScan v31 (Punta et al. 2012) and those with the target domains (Table S5) were selected for annotation with GeMoMa. GeMoMa annotations were filtered using the GeMoMa Annotation filter (GAF), either retaining only complete gene models or all predictions (i.e. parameter settings: -r 0 -e 0). The Exonerate, GeMoMa (i.e. all predictions) and GAF gene models, as well as the mapped worker RNAseq reads (HISAT2 (Kim et al. 2015)) were used as evidence tracks for further manual annotation using Web Apollo v2.0.8. For each gene family, manual annotations in *C. levior* and associated orthologs in the reference species were aligned with MAFFT v7.123b (parameter settings: --maxiterate 1000 ‑‑localpair). Protein trees were obtained with FastTree (Price et al. 2009) (parameter settings: --pseudo). Alignments were visually checked to identify potentially fragmented annotations, which were further curated in Web Apollo. In the second round, Exonerate and GeMoMa were run again, now using the manual annotations from round one as queries. Predictions were used as evidence track in Web Apollo to manually annotate gene models that were missed in the first round. The second round yielded an additional 14 gene models, including 5 ORs, 4 ELOs, 1 OBP, 1 CSP, 1 DESAT and 2 GRs. The manually annotated protein predictions were functionally annotated with PfamScan and only those that contained the defining domains (Table S5) were retained (Table S6).

**Table S4:** Reference species set, and versions used for GeMoMa annotations

| **Abbr.** | **Species** | **genome.fa** | **genome.gff** |
| --- | --- | --- | --- |
| aech | *Acromyrmex echinatior* | Aech_2.0 | aech_OGSv3.8 |
| acep | *Atta cephalotes* | Acep_1.0 | acep_OGSv1.2 |
| cobs | *Cardiocondyla obscurior* | Cobs_1.4 | Cobs_1.4 |
| cflo | *Camponotus floridanus* | Cflo_3.3 | cflo_OGSv3.3 |
| hsal | *Harpegnathos saltator* | Hsal_3.3 | hsal_OGSv3.3 |
| lhum | *Linepithema humile* | Lhum_1.0 | lhum_OGSv1.2 |
| pbar | *Pogonomyrmex barbatus* | Pbar_1.0 | pbar_OGSv1. |
| tlon | *Temnothorax longispinosus* | tlon_1.0 | tlon_1.0 |

**Table S5**: Target gene families and associated Pfam domains used for identification

| **gene family** | **abbr.** | **domain** |
| --- | --- | --- |
| odorant receptors | OR | 7tm_6 |
| gustatory receptors | GR | 7tm_7 + Trehalose_recp |
| elongases | ELO | ELO |
| desaturase | DESAT | FA_desaturase |
| odorant binding proteins | OBP | PBP_GOBP |
| chemosensory proteins | CSP | OS-D |

M8: Annotation of elongases and desaturases in other Hymenoptera

We used a reference set of nine high-quality insect genome assemblies (Table S7) to annotate elongases and desaturases in the available hymenopteran genomes (Table S8). To build the databases for the two gene families, we ran PfamScan on the protein sequences and subsequently filtered the output for those containing the defining ELO and DESAT domains (Table S5). The resulting target databases were then used to blast against the remaining 43 annotated Hymenoptera protein sets (Table S8) that were not contained within the reference set with blastp v2.5.1 (e-value < 1e-03). On proteins for which blast hits were found, we ran PfamScan to confirm the presence of the target domain. We then filtered the resulting sets of ELO and DESAT genes for identical duplicates to correct for putative assembly mistakes. We tested for significant differences in ELO and DESAT gene number between different families of Hymenoptera with a one-way ANOVA followed by Tukey’s HSD, using R v3.5.2 (R Core 2018). Results can be seen in Table S9.

**Table S6**: Total number of target genes per family

| **gene family** | **total count** |
| --- | --- |
| OR | 282 |
| GR | 57 |
| ELO | 23 |
| DESAT | 25 |
| OBP | 14 |
| CSP | 17 |

| **species** | **genome version** | **GeneBank Assembly Accession** |
| --- | --- | --- |
| *Apis mellifera* | Amel_4.5 | GCF_000002195.4 |
| *Drosophila melanogaster* | Dmel_6plusISO1MT | GCA_000001215.4 |
| *Bombus impatiens* | Bimp_2.0 | GCF_000188095.1 |
| *Tribolium castaneum* | Tcas_5.2 | GCF_000002335.3 |
| *Musca domestica* | Mdom_2.0.2 | GCF_000371365.1 |
| *Crematogaster levior* | Clev_1.0 | ERS3409635 |
| *Acromyrmex echinatior* | Aech_3.9 | GCF_000204515.1 |
| *Nasonia vitripennis* | Nvit_2.1 | GCF_000002325.3 |
| *Copidosoma floridanum* | Coflo_2.0 | GCF_000648655.2 |

**Table S7:** Reference species set for identification of ELO and DESAT genes

**Table S8:** Overview of the different protein sets used in ELO and DESAT comparison. Species marked with an asterisk were used as reference species

| **Family** | **Species** | **abbr.** | **genome version** | **#ELO** | **#DESAT** |
| --- | --- | --- | --- | --- | --- |
| ant | *Acromyrmex echinatior** | aech | Aech_3.9 | 20 | 18 |
| ant | *Atta cephalotes* | acep | Acep_1.0 | 18 | 11 |
| ant | *Atta colombica* | acol | Acol_1.0 | 12 | 12 |
| ant | *Camponotus floridanus* | cflo | Caflo_1.0 | 20 | 13 |
| ant | *Cyphomyrmex costatus* | ccos | Ccos_1.0 | 13 | 16 |
| ant | *Cardiocondyla obscurior* | cobs | Cobs_1.4 | 12 | 6 |
| ant | *Crematogaster levior** | clev | Clev_1.0 | 23 | 25 |
| ant | *Dinoponera quadriceps* | dqua | Dqua_1.0 | 20 | 11 |
| ant | *Harpegnathos saltator* | hsal | Hsal_1.0 | 19 | 14 |
| ant | *Lasius niger* | lnig | Lnig_1.0 | 9 | 9 |
| ant | *Linepithema humile* | lhum | Lhum_04 | 33 | 15 |
| ant | *Monomorium pharaonis* | mpha | Mpha_2.0 | 20 | 12 |
| ant | *Ooceraea biroi* | obir | Obir_1.0 | 14 | 8 |
| ant | *Pogonomyrmex barbatus* | pbar | Pbar_03 | 17 | 12 |
| ant | *Pseudomyrmex gracilis* | pgra | Pgra_1.0 | 36 | 25 |
| ant | *Solenopsis invicta* | sinv | Sinv_Si_gnG | 29 | 15 |
| ant | *Trachymyrmex cornetzi* | tcor | Tcor_1.0 | 22 | 14 |
| ant | *Trachymyrmex septentrionalis* | tsep | Tsep_1.0 | 15 | 14 |
| ant | *Trachymyrmex zeteki* | tzet | Tzet_1.0 | 18 | 11 |
| ant | *Vollenhovia emeryi* | veme | Veme_1.0 | 37 | 17 |
| ant | *Wasmannia auropunctata* | waur | Waur_1.0 | 25 | 9 |
| bee | *Apis cerana* | acer | Acer_2.0 | 17 | 7 |
| bee | *Apis dorsata* | ador | Ador_1.3 | 13 | 11 |
| bee | *Apis florea* | aflo | Aflo_1.0 | 17 | 8 |
| bee | *Apis mellifera** | amel | Amel_4.5 | 17 | 7 |
| bee | *Bombus impatiens** | bimp | Bimp_2.0 | 21 | 9 |
| bee | *Bombus terrestris* | bter | Bter_1.0 | 21 | 7 |
| bee | *Ceratina calcarata* | ccal | Ccal_1.0 | 20 | 6 |
| bee | *Dufourea novaengliae* | dnov | Dnov_1.0 | 15 | 9 |
| bee | *Eufriesea mexicana* | emex | Emex_1.0 | 16 | 10 |
| bee | *Euglossa dilemma* | edil | Edil_1.0 | 15 | 5 |
| bee | *Habropoda laboriosa* | hlab | Hlab_1.0 | 13 | 6 |
| bee | *Lasioglossum albipes* | lalb | Lalb_5.4 | 16 | 12 |
| bee | *Melipona quadrifasciata* | mqua | Mqua_1.0 | 19 | 4 |
| bee | *Megachile rotundata* | mrot | Mrot_1.0 | 17 | 16 |
| wasp | *Copidosoma floridanum** | coflo | Coflo_2.0 | 12 | 18 |
| wasp | *Diachasma alloeum* | dall | Dall_1.0 | 13 | 9 |
| wasp | *Fopius arisanus* | fari | Fari_1.0 | 12 | 12 |
| wasp | *Microplitis demolitor* | mdem | Mdem_2.0 | 13 | 12 |
| wasp | *Nasonia vitripennis** | nvit | Nvit_2.1 | 13 | 18 |
| wasp | *Polistes canadensis* | pcan | Pcan_1.0 | 14 | 9 |
| wasp | *Polistes dominula* | pdom | Pdom_r1.2 | 15 | 9 |
| wasp | *Trichogramma pretiosum* | tpre | Tpre_2.0 | 14 | 13 |
| wasp | *Trichomalopsis sarcophagae* | tsar | Tsar_1.0 | 9 | 11 |
| sawfly | *Athalia rosae* | aros | Aros_1.0 | 10 | 17 |
| sawfly | *Cephus cinctus* | ccin | Ccin_1.0 | 16 | 13 |
| sawfly | *Neodiprion lecontei* | nlec | Nlec_1.0 | 18 | 20 |
| sawfly | *Orussus abietinus* | oabi | Oabi_2.0 | 12 | 11 |

**Table S9**: Results of a one-way ANOVA followed by Tukey’s HSD comparing total ELO and DESAT numbers between different families of Hymenoptera. Bold type indicates significance (p < 0.05).

|  |  | **Ant** | | | | **Bee** | | | | **Wasp** | | | |
| --- | --- | --- | --- | --- | --- | --- | --- | --- | --- | --- | --- | --- | --- |
|  |  | diff | lwr | upr | padj | diff | lwr | upr | padj | diff | lwr | upr | padj |
| **ELO** | **Ant** |  |  |  |  |  |  |  |  |  |  |  |  |
|  | **Bee** | -3.64 | -8.75 | 1.47 | 0.24 |  |  |  |  |  |  |  |  |
|  | **Wasp** | **-7.79** | **-13.7** | **-1.89** | **0.005** | -4.15 | 10.48 | 2.18 | 0.31 |  |  |  |  |
|  | **Sawfly** | -6.57 | -14.7 | 1.51 | 0.15 | -2.93 | -11.3 | 5.47 | 0.79 | -1.22 | -10.1 | 7.68 | 0.98 |
| **DESAT** | **Ant** |  |  |  |  |  |  |  |  |  |  |  |  |
|  | **Bee** | **-5.31** | **-9.08** | **-1.54** | **0.003** |  |  |  |  |  |  |  |  |
|  | **Wasp** | -1.33 | -5.69 | 3.03 | 0.85 | 3.98 | -0.7 | 8.65 | 0.12 |  |  |  |  |
|  | **Sawfly** | 1.58 | -4.38 | 7.55 | 0.89 | **6.89** | **0.69** | **13.1** | **0.024** | -2.92 | -9.49 | 3.66 | 0.64 |

**Figure S1:** Proportion of different repeat classes in the C. levior genome


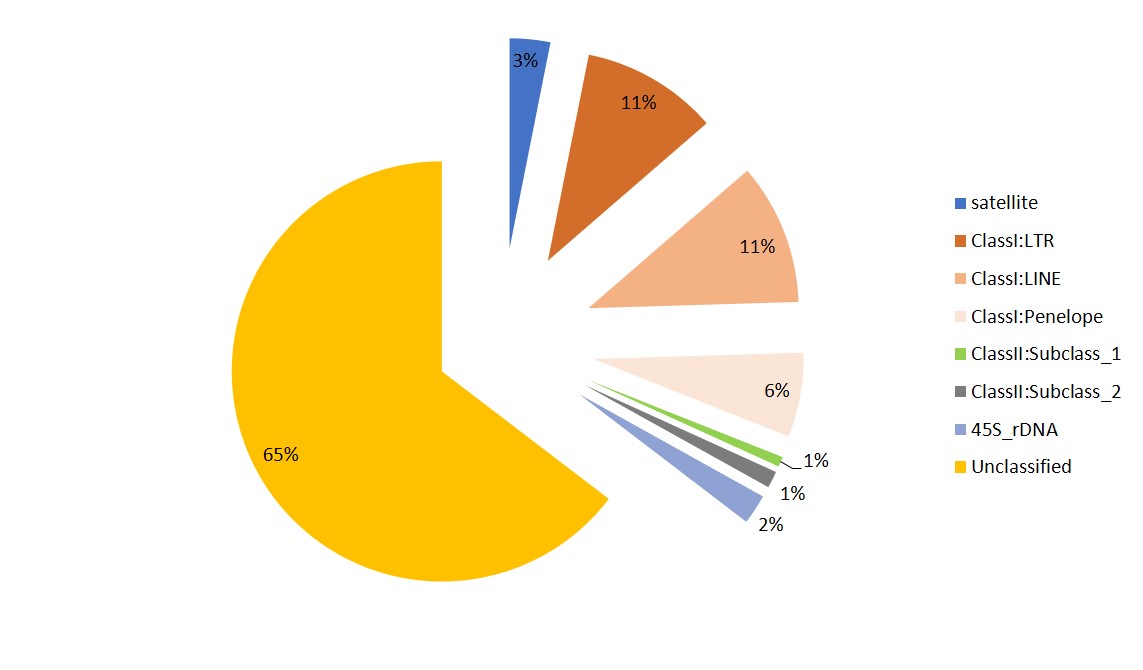


| ***Wasmannia auropunctata*** | ***Vollenhovia emeryi*** | ***Trachymyrmex zeteki*** | ***Trachymyrmex septentrionalis*** | ***Trachymyrmex cornetzi*** | ***Solenopsis invicta*** | ***Pseudomyrmex gracilis*** | ***Pogonomyrmex barbatus*** | ***Monomorium pharaonis*** | ***Linepithema humile*** | ***Lasius niger*** | ***Harpegnathos saltator*** | ***Dinoponera quadriceps*** | ***Cyphomyrmex costatus*** | ***Ooceraea biroi*** | ***Crematogaster levior A*** | ***Cardiocondyla obscurior*** | ***Camponotus floridanus*** | ***Atta colombica*** | ***Atta cephalotes*** | ***Acromyrmex echinatior*** | **Species** |
| --- | --- | --- | --- | --- | --- | --- | --- | --- | --- | --- | --- | --- | --- | --- | --- | --- | --- | --- | --- | --- | --- |
|  |  | 294.8 | 294.4 | 396.1 | 463 - 753.3 | 391.20 | 245 - 280 |  | 254.28 |  | 330.00 |  | 318.5 |  | 409.96 |  | 303 - 323 | 303.0 | 303.18 | 335.0 | **Estimated Genome size [Mbp]** |
| 324.12 | 287.9 | 268.0 | 291.7 | 369.4 | 399.0 | 282.8 | 235.6 | 258.0 | 219.5 | 236.2 | 294.5 | 259,7 | 300. 3 | 212.8 | 326.2 | 177.9 | 232.7 | 291.3 | 317.7 | 295.9 | **Assembly Length [Mbp]** |
| 77,788 | 13,258 | 4,623 | 5,836 | 19,761 | 66,904 | 6,556 | 4,645 | 12,136 | 3,030 | 36,804 | 8,893 | 14,123 | 15,379 | 4,579 | 1,523 | 1,854 | 10,791 | 1,550 | 2,835 | 4,339 | **# Scaffolds** |
| 1,175,369 | 1,346,088 | 1,333,945 | 2,520,094 | 760,749 | 621,039 | 317,681 | 819,605 | 75,377 | 1,402,257 | 17,057 | 601,965 | 1,361,239 | 1,159,032 | 1,350,650 | 383,244 | 3,105,814 | 451,320 | 2,037,154 | 5,154,485 | 1,110,580 | **N50** |
| 15,458 | 14,870 | 15,530 | 15,575 | 19,827 | 16,569 | 16,069 | 17,177 | 13,616 | 16,123 | 19,989 | 18,564 | 13,688 | 16,468 | 17,263 | 17,855 | 17,552 | 17,064 | 14,345 | 18,093 | 17,278 | **# Genes** |
| 6241 | 6283 | 6240 | 6288 | 6242 | 4146 | 6264 | 5711 | 6261 | 5914 | 4920 | 5940 | 6269 | 6230 | 6019 | 5652 | 6084 | 6028 | 6265 | 5606 | 5945 | **# Dogma CDAs** |
| 97.90% | 98.60% | 97.90% | 98.70% | 97.90% | 65.10% | 98.30% | 89.60% | 98.20% | 92.80% | 77.20% | 93.20% | 98.40% | 97.80% | 94.50% | 88.70% | 95.50% | 94.60% | 98.30% | 88.00% | 93.30% | **Dogma [%]** |
|  |  | 24.90% | 23.90% | 42.10% | 23.00% | 41.00% | 11.60% |  | 16.50% | 1.40% | 26.90% | 6.00% | 34.00% | 13.80% | 10.50% |  | 15.10% | 31.80% | 25.00% | 27.70% | **Repeats [%]**  **of assembly** |
|  | Mikheyev & Linksvayer 2015 | Nygaard et al. 2016 | Nygaard et al. 2016 | Nygaard et al. 2016 | Wurm et al. 2011 | Rubin et al. 2016 | Chris R Smith et al. 2011 | Mikheyev & Linksvayer 2015 | Smith et al. 2011 | Konorov et al. 2017 | Bonasio et al. 2010 | Patalano et al. 2015 | Nygaard et al. 2016 | Oxley et al. 2014 |  | Schrader et al. 2014 | Bonasio et al. 2010 | Nygaard et al. 2016 | Suen et al. 2011 | Nygaard et al. 2011 | **Publication** |
|  |  | Nygaard et al. 2016 | Nygaard et al. 2016 | Nygaard et al. 2016 | Gadau et al. 2012 | Ardila-Garcia et al. 2010 | Chris R Smith et al. 2011 |  | Gadau et al. 2012 |  | Gadau et al. 2012 |  | Nygaard et al. 2016 |  |  |  | Gadau et al. 2012 | Tsutsui et al. 2008 | Gadau et al. 2012 | Gadau et al. 2012 | **Source Genome size** |

**Table S10**: Overview of annotated ant genomes and according assembly statistics. Assembly statistics were analysed using own summary pipeline. Values might therefore differ from respective publications. For analysed genome versions refer to Table S8.

**Table S11:** Backmapping rates of different read sets on the final assembly

| **Read type** | **Percentage of mapped reads** |  |  |
| --- | --- | --- | --- |
| Illumina reads | 97.31% | |  |
| Illumina contigs | 76.90% | |  |
| PacBio reads | 97.49% | |  |
| PacBio reads corrected | 96.27% | |  |
| MinION reads | 96.89% | |  |
|  |  | |  |

**Table S12:** Percentage of the genome that is only covered by one type of sequencing technique

| **Read type** |  | **Percentage of genome** |
| --- | --- | --- |
| Illumina contigs | | 1.05% |
| PacBio | | 2.33% |
| PacBio uncorrected | | 1.31% |
| MinION | | 2.42% |

# References

Andrews S. 2010. FastQC: a quality control tool for high throughput sequence data. http//www.bioinformatics.babraham.ac.uk/ Proj.

Ardila-Garcia AM, Umphrey GJ, Gregory TR. 2010. An expansion of the genome size dataset for the insect order Hymenoptera, with a first test of parasitism and eusociality as possible constraints. Insect Mol. Biol. 19:337–346. doi: 10.1111/j.1365-2583.2010.00992.x.

Bankevich A et al. 2012. SPAdes: A New Genome Assembly Algorithm and Its Applications to Single-Cell Sequencing. J. Comput. Biol. 19:455–477. doi: 10.1089/cmb.2012.0021.

Boetzer M, Pirovano W. 2014. SSPACE-LongRead: scaffolding bacterial draft genomes using long read sequence information. BMC Bioinformatics. 15:1–9.

Bolger AM, Lohse M, Usadel B. 2014. Trimmomatic: A flexible trimmer for Illumina sequence data. Bioinformatics. 30:2114–2120. doi: 10.1093/bioinformatics/btu170.

Bonasio R et al. 2010. Genomic Comparison of the Ants *Camponotus floridanus* and *Harpegnathos saltator*. Science (80-. ). 329:1068–1071. doi: 10.1126/science.1192428.

Dohmen E, Kremer LPM, Bornberg-Bauer E, Kemena C. 2016. DOGMA: Domain-based transcriptome and proteome quality assessment. Bioinformatics. 32:2577–2581. doi: 10.1093/bioinformatics/btw231.

Doležel J, Bartoš J, Voglmayr H, Greilhuber J. 2003. Nuclear DNA content and genome size of trout and human. Cytom. Part A. 51 (2):127–128. doi: 10.1002/cyto.a.10013.

Doležel J, Doleželová M, Novák FJ. 1994. Flow cytometric estimation of nuclear DNA amount in diploid bananas (*Musa acuminata* and *M. balbisiana*). Biol. Plant. 36:351–357.

El-Gebali S et al. 2019. The Pfam protein families database in 2019. Nucleic Acids Res. 47:D427–D432. doi: 10.1093/nar/gky995.

Gadau J et al. 2012. The genomic impact of 100 million years of social evolution in seven ant species. Trends Genet. 28:14–21. doi: 10.1016/j.tig.2011.08.005.

Grabherr MG et al. 2013. Trinity: reconstructing a full-length transcriptome without a genome from RNA-Seq data. Nat. Biotechnol. 29:644–652. doi: 10.1038/nbt.1883.Trinity.

Greilhuber J, Doležel J, Lysák MA, Bennett MD. 2005. The Origin , Evolution and Proposed Stabilization of the Terms ‘Genome Size’ and ‘C-Value’ to Describe Nuclear DNA Contents. Ann. Bot. 255–260. doi: 10.1093/aob/mci019.

Hackl T, Hedrich R, Schultz J, Förster F. 2014. Proovread: Large-scale high-accuracy PacBio correction through iterative short read consensus. Bioinformatics. 30:3004–3011. doi: 10.1093/bioinformatics/btu392.

Hahn C, Bachmann L, Chevreux B. 2013. Reconstructing mitochondrial genomes directly from genomic next-generation sequencing reads — a baiting and iterative mapping approach. Nucleic Acids Res. 41. doi: 10.1093/nar/gkt371.

Holt C, Yandell M. 2011. MAKER2: an annotation pipeline and genome-database management tool for second-generation genome projects. BMC Bioinformatics. 12:491. doi: 10.1186/1471-2105-12-491.

Kaur R et al. 2019. Ant behaviour and brain gene expression of defending hosts depend on the ecological success of the intruding social parasite. Philos. Trans. R. Soc. B Biol. Sci. 374. doi: 10.1098/rstb.2018.0192.

Keilwagen J et al. 2016. Using intron position conservation for homology-based gene prediction. Nucleic Acids Res. 44. doi: 10.1093/nar/gkw092.

Kim D, Langmead B, Salzberg SL. 2015. HISAT: a fast spliced aligner with low memory requirements. Nat. Methods. 12. doi: 10.1038/nmeth.3317.

Konorov EA et al. 2017. Genomic exaptation enables *Lasius niger* adaptation to urban environments. BMC Evol. Biol. 17:39. doi: 10.1186/s12862-016-0867-x.

Korf I. 2004. Gene finding in novel genomes. BMC Bioinformatics. 9:1–9.

Lee E et al. 2013. Web Apollo: a web-based genomic annotation editing platform. Genome Biol. 14.

Lomsadze A, Ter-Hovhannisyan V, Chernoff YO, Borodovsky M. 2005. Gene identification in novel eukaryotic genomes by self-training algorithm. Nucleic Acids Res. 33:6494–6506. doi: 10.1093/nar/gki937.

Mikheyev AS, Linksvayer TA. 2015. Genes associated with ant social behavior show distinct transcriptional and evolutionary patterns. Elife. 2015:1–17. doi: 10.7554/eLife.04775.

Nygaard S et al. 2016. Reciprocal genomic evolution in the ant-fungus agricultural symbiosis. Nat. Commun. 7:1–9. doi: 10.1038/ncomms12233.

Nygaard S et al. 2011. The genome of the leaf-cutting ant *Acromyrmex echinatior* suggests key adaptations to advanced social life and fungus farming. Genome Res. 21:1–10. doi: 10.1101/gr.121392.111.

Otto F. 1990. DAPI Staining of Fixed Cells for High-Resolution Flow Cytometry of Nuclear DNA. In: Darzynkiewickz Z, Crissman HA, eds. Methods Cell Biol. 33:105–110.

Oxley PR et al. 2014. The genome of the Clonal raider ant *Cerapachys biroi*. Curr. Biol. 24:451–458. doi: 10.1016/j.cub.2014.01.018.

Parra G, Bradnam K, Korf I. 2007. CEGMA: a pipeline to accurately annotate core genes in eukaryotic genomes. Bioinformatics. 23:1061–1067. doi: 10.1093/bioinformatics/btm071.

Patalano S et al. 2015. Molecular signatures of plastic phenotypes in two eusocial insect species with simple societies. Proc. Natl. Acad. Sci. 112:13970–13975. doi: 10.1073/pnas.1515937112.

Price MN, Dehal PS, Arkin AP. 2009. FastTree: Computing Large Minimum Evolution Trees with Profiles instead of a Distance Matrix. Mol. Bol. Evol. 26:1641–1650. doi: 10.1093/molbev/msp077.

Punta M et al. 2012. The Pfam protein families database. Nucleic Acids Res. 40:290–301. doi: 10.1093/nar/gkr1065.

R Core T. 2018. R Foundation for Statistical Computing; Vienna, Austria: 2014. R A Lang. Environ. Stat. Comput. 2013.

Rubin BER et al. 2016. Comparative genomics reveals convergent rates of evolution in ant–plant mutualisms. Nat. Commun. 7:12679. doi: 10.1038/ncomms12679.

Schell T et al. 2017. An Annotated Draft Genome for *Radix auricularia* (Gastropoda, Mollusca). Genome Biol. Evol. 9:585–592. doi: 10.1093/gbe/evx032.

Schrader L et al. 2014. Transposable element islands facilitate adaptation to novel environments in an invasive species. Nat. Commun. 5:5495. doi: 10.1038/ncomms6495.

Simão FA, Waterhouse RM, Ioannidis P, Kriventseva E V, Zdobnov EM. 2015. Genome analysis BUSCO: assessing genome assembly and annotation completeness with single-copy orthologs. Bioinformatics. 31:3210–3212. doi: 10.1093/bioinformatics/btv351.

Slater GSC, Birney E. 2005. Automated generation of heuristics for biological sequence comparison. BMC Bioinformatics. 11:1–11. doi: 10.1186/1471-2105-6-31.

Smit AFA, Hubley R, Green P. 2008. RepeatModeler Open-1.0. 2008–2015. Inst. Syst. Biol. Seattle, WA, USA.

Smith Christopher D et al. 2011. Draft genome of the globally widespread and invasive Argentine ant (*Linepithema humile*). Proc. Natl. Acad. Sci. U. S. A. 108:5673–5678. doi: 10.1073/pnas.1008617108.

Smith Chris R et al. 2011. Draft genome of the red harvester ant *Pogonomyrmex barbatus*. Proc. Natl. Acad. Sci. U. S. A. 108:5667–72. doi: 10.1073/pnas.1007901108.

Stanke M et al. 2006. AUGUSTUS: ab initio prediction of alternative transcripts. Nucleic Acids Res. 34:W435–W439. doi: 10.1093/nar/gkl200.

Suen G et al. 2011. The genome sequence of the leaf-cutter ant *Atta cephalotes* reveals insights into its obligate symbiotic lifestyle. PLoS Genet. 7. doi: 10.1371/journal.pgen.1002007.

Tsutsui ND, Suarez A V, Spagna JC, Johnston JS. 2008. The evolution of genome size in ants. BMC Evol. Biol. 8:64. doi: 10.1186/1471-2148-8-64.

Urban JM, Bliss J, Lawrence CE, Gerbi SA. 2015. Sequencing ultra-long DNA molecules with the Oxford Nanopore MinION. BioRxiv Prepr. doi: 10.1002/dvdy.

Waldvogel AM et al. 2018. The genomic footprint of climate adaptation in Chironomus riparius. Mol. Ecol. 27:1439–1456. doi: 10.1111/mec.14543.

Wingett SW, Andrews S, Hamilton RS. 2018. FastQ Screen: A tool for multi-genome mapping and quality control. F1000Research. 1–12. doi: 10.12688/f1000research.15931.1.

Wurm Y et al. 2011. The genome of the fire ant *Solenopsis invicta*. Proc. Natl. Acad. Sci. 108:5679–5684. doi: 10.1073/pnas.1009690108.
